# Applying Blockchain Technology and the Internet of Things to Improve the Data Reliability for Livestock Insurance

**DOI:** 10.3390/s23146290

**Published:** 2023-07-11

**Authors:** Lihua Shen, Zhibin Zhang, Youmei Zhou, Yingying Xu

**Affiliations:** 1 Zhijiang College, Zhejiang University of Technology, Shaoxing 312030, China; shenlihua@zzjc.edu.cn; 2 School of Management, Zhejiang University of Technology, Hangzhou 310014, China; 2112104215@zjut.edu.cn; 3 Department of Landscape Architecture, Tongji University, Shanghai 200092, China; 4 School of Humanities and Social Science, Beihang University, Beijing 100191, China; yingxu21@buaa.edu.cn

**Keywords:** blockchain, Internet of Things (IoT), livestock insurance, smart contract

## Abstract

Animal husbandry is a vital sector in China’s agriculture sector, contributing to over one-third of its agricultural output, and more than 40% of farmers’ income. However, this industry is vulnerable to risks arising from production and operation, such as disease outbreaks, natural disasters, and market fluctuations. Livestock insurance can help mitigate these risks, but the lack of reliable data on shed environments has hindered its effectiveness. The objective of this study is to propose a livestock shed environmental regulatory platform that utilizes blockchain and the Internet of Things to ensure data authenticity, real-time monitoring, and transparency in the regulatory process. The platform also automates the insurance process, reducing costs and improving efficiency. The proposed platform employs blockchain to ensure data authenticity and devices to monitor and collect real-time environmental data. It also utilizes smart contracts to automate the insurance process, from negotiating and signing contracts to making insurance claims. The system’s design rationale, architecture, and implementation are detailed. The proposed platform has been implemented and currently manages over 300,000 livestock animals with more than 350,000 insurance contracts signed. The use of blockchain and the Internet of Things has ensured data authenticity, real-time monitoring, and transparency in the regulatory process, while the automation of the insurance process has reduced costs and improved efficiency. The proposed livestock shed environmental regulatory platform has the potential to improve the effectiveness of livestock insurance in China by addressing the critical issue of data reliability. The use of blockchain and the Internet of Things has enabled real-time monitoring, data authenticity, and transparency in the regulatory process, while the automation of the insurance process has improved efficiency and reduced costs. This platform could serve as a model for other countries looking to improve the effectiveness of their livestock insurance programs.

## 1. Introduction

Livestock insurance can effectively prevent the risks arising from the production and operation of livestock, which is of great significance to stabilizing the development of the livestock industry in various countries around the world [1,2]. According to the survey conducted by the Food and Agriculture Organization (FAO) on agricultural insurance in the Asia-Pacific region in 2017, 88% of the countries in Asia that carry out agricultural insurance have introduced livestock insurance schemes, among which 43% of the countries provide different forms of government subsidies to support livestock insurance [3]. In China, the livestock industry is developing rapidly, and over 34% of the total agricultural output is attributed to the livestock industry [4,5]. Therefore, China’s livestock industry has played a vital role in ensuring stable food prices and promoting farmers’ incomes; and in some provinces, livestock has become a pillar industry of the rural economy and a major source of farmers’ income. At present, most provinces in China have carried out livestock insurance. Among them, the insurance for sows, dairy cows, and fattening pigs has been expanded to all provinces in China, and the insurance schemes for yaks and Tibetan sheep have been carried out in Sichuan, Qinghai, and Tibet [6]. In order to encourage these herders to ensure their livestock, the government usually offers subsidies [7].

However, livestock insurance is still in its initial stage in China [8,9], which is manifested through several aspects. For example, the operation and management of the traditional livestock industry relies on a large number of people, usually resulting in low productivity. Moreover, the insurance coverage on livestock assets is insufficient due to the complex procedures of insurance applications and claim requests. In addition, the market of livestock products is an opaque one, and the supply and demand are always in cyclical fluctuations, making it difficult for herders to obtain accurate data for market prediction and increasing the risk of breeding. The prevention of insurance fraud is still a top priority for the insurance industry [10], and subsidy fraud is a somewhat serious challenge for regulatory authorities [7]. These problems have greatly hindered the widespread adoption of livestock insurance [11].

Blockchain technology (BCT) has received increasing attention since 2008 [12]. A number of BCT-driven applications indicate that BCT significantly benefits financial, social, and health contexts [13,14,15,16,17]. e.g., Vahdati et al. (2018) proposed a self-organized framework for insurance based on IoT and blockchain, which enables real-time data collection and analysis for risk assessment [18]. Oham et al. (2018) developed a blockchain-based framework for auto-insurance claims and adjudication, which improves the efficiency and transparency of claims processing [19]. Xiao et al. (2020) presented a case study on fine-grained transportation insurance [20], which demonstrated the effectiveness of using IoT and Blockchain for risk assessment and claims processing. The integration of IoT and Blockchain in insurance and healthcare also brings new opportunities and challenges. The primary objective of adopting blockchain is to reshape the claims-handling process and payments, thereby reducing the possibility of fraudulent claims. Moreover, applying BCT can remove intermediaries, which are usually the brokers of insurers dealing with consumers. The major objectives of the above-mentioned solutions are: (1) to speed up the handling process of the supply chain; (2) to ensure tamper-proof transaction records; and (3) to make the data visible and accessible only to participants involved.

In this study, we propose a BCT and Internet of Things-based solution to provide a regulatory platform for the livestock insurance management process. Each livestock is assigned a unique digital identity through an RFID ear tag, which enables a one-to-one mapping between individual livestock and its digital identity in the system. The proposed system allows government regulators and financial institutions (such as insurance companies, banks, etc.) to track the full procedures of livestock management at any time. Thus, enhancing the efficiency and transparency of livestock insurance can significantly encourage the adoption of livestock insurance [21]. Compared to previous studies, our proposed solution utilizes BCT and IoT to address the challenges of data reliability and transparency in the livestock insurance industry.

The key contributions in this paper are summarized as follows. We propose a BCT-based information regulatory platform for livestock insurance. The data regarding the whole cycle of livestock breeding, which is provided by the platform, is authentic, reliable, and easily verified by each participant in the blockchain network. This significantly reduces the risks of insurance fraud and subsidy fraud.

By using IoT technology, we developed a real-time data detection system. It is capable of the following: (a) accurately identifying and precisely tracking livestock; (b) monitoring and managing the ranch environment and livestock sheds. With this system, traditional human-led livestock management is upgraded to intelligence-driven management. We use the smart contract to automate all the business processes of livestock insurance (i.e., insurance clause negotiation, insurance contract generation, and insurance claim settlement), which could improve efficiency and encourage the adoption of livestock insurance on a large scale.

## 2. Background

### 2.1. Blockchain Technology

The BCT underlying Bitcoin [12] is a typical implementation of a fully decentralized shared ledger, which aims to ensure the immutability and authenticity of transaction data and public access. Generally, BCT is the collaborative combination of various technologies, such as a peer-to-peer network, consensus mechanism, and smart contract, to construct a new data organization and management solution for the application context of multiple participants involved. Different from a single source of truth, BCT is in essence a distributed ledger technology based on the principle of cryptography. Specifically, blockchain is a data structure, which contains data blocks in a chain in chronological order and cryptographically guarantees the immutability of data.

BCT has brought promising opportunities for the global economy due to the following reasons [22,23]. Currently, the private and public sectors pay much attention to certified blockchains to take advantage of their decentralized design [24,25]. Azzaoui et al. (2020) present an analytics framework-Block5GIntell depicting the applications of blockchain and AI on 5G networks [8]. Uddin et al. (2021) survey the state-of-the-art advances in blockchain for IoT with applications in the fields of smart cities, intelligent transport, and eHealth [10,11,12,13,14]. A number of works study the role of the emerging blockchain technology in the green IoT ecosystem and exploit the critical factors for building a green IoT ecosystem, with the features enabled by blockchain technology [9,26,27].

### 2.2. Blockchain and IoT in Insurance Industry

There are many practical cases of adopting BCT in the insurance industry. For insurance, AON, a global risk management company developed a blockchain-based agricultural insurance policy for farmers. American International Group, Inc. has also proposed a blockchain-based operation platform to provide nationwide insurance services [28]. The processing of claims is driven by smart contracts and involves no manual intervention [29,30,31,32]. A number of works regarding transportation insurance are proposed with the engagement of blockchain and Internet of Things (IoT) technologies for [33,34,35,36,37,38]. e.g., Singer [16] argues that BCT can help the insurance industry to address the issue of fraudulent claims. Chen et al. (2020) proposed a BCT-based solution to address the issue of car milometers in the used-car industry [23].

Healthcare systems generate a vast amount of data, and the integration of IoT and blockchain can be used to secure, share, and exchange this data [18,26,39,40,41,42]. e.g., Nair and Bhagat (2020) proposed a blockchain-based approach for healthcare information exchange [39]. Satamraju (2020) developed a proof of concept for scalable integration of IoT and blockchain in healthcare. Xiao et al. (2020) proposed a case study and cyberinfrastructure solution for fine-grained transportation insurance [20].

In summary, IoT and Blockchain have shown great potential for transforming the insurance and healthcare sectors. However, there are still challenges that need to be addressed, such as data privacy and security, interoperability, and regulatory compliance. These existing studies only focused on livestock traceability or health management, without full coverage of the livestock insurance management process.

## 3. Materials and Methods

This study proposes a decentralized regulatory system, which aims to comprehensively improve the capability of livestock management and data governance. The objectives of the proposed solution are: (a) to establish a unified identity management system for livestock assets; (b) to establish a mechanism to track and retrospect the whole-life cycle of livestock; and (c) to establish a risk management system of livestock breeding while providing the capability of risk control for insurance and financing businesses.

**Figure 1:**
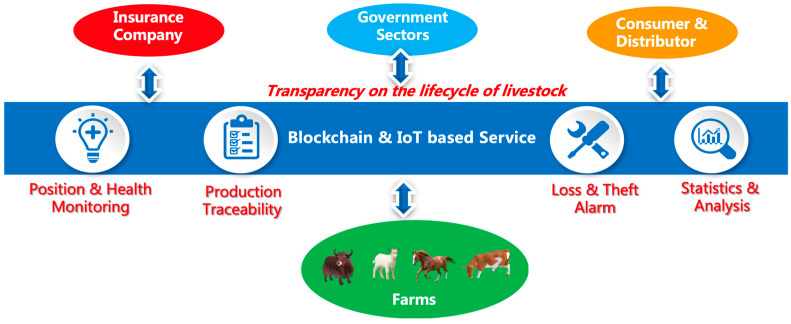
The simplified business model of livestock insurance.

Figure 1 presents the simplified business logic as the system provides services to different users. The quarantine department is mainly concerned with vaccine quarantine verification and health data checking. Meanwhile, the agricultural regulatory department is concerned with the farming process specification. The environmental protection department is concerned about the environmental issues during the breeding process (such as breeding waste disposal). However, the main concern for farmers is livestock breeding information. Slaughterhouses need to be able to assist in livestock slaughter management with the help of a system, and distributors need the slaughter information. Comprehensive information regarding the health condition of the livestock insured is required by insurance agencies, while financial institutions focus on information collection on farmers’ assets and livestock breeding to make reasonable risk assessments.

### 3.1. Architecture

**Figure 2:**
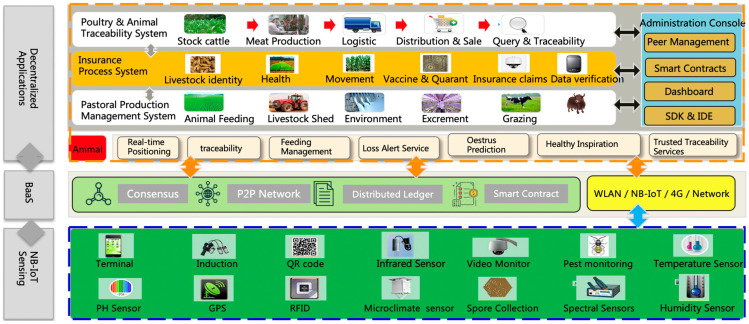
The overview of the proposed system architecture.

The integrated system architecture (as shown in Figure 2) consists of a decentralized application, blockchain infrastructure, and IoT intelligent sensing layers, and we employ various kinds of programming languages (Python, Nodejs, Java, HTML 5, etc.) to develop this system.

The intelligent sensing layer adopts the narrowband Internet of Things technology to integrate sensors (such as temperature sensors and humidity sensors) and low-energy devices. The narrowband Internet of Things network features large capacity, low cost, and a 5-year life [43,44,45,46]. The intelligent sensing layer is capable of swiftly transmitting the data collected by the sensors to the backend server through wireless transmission. The data collection module provides data collection, which can dock IoT protocols of different manufacturers. It periodically accesses devices, parses the data packets, and uploads the data to the database. The device management module realizes the functions of adding, modifying, importing, and exporting devices. The security management module ensures the all-around data security of the platform. It provides a security link authentication code for the device side, and when the device uploads data to the network.

We use consortium blockchain to build the underlying architecture. The participants include farmers, insurance companies, IoT services, and governmental departments (e.g., the environment protection bureau). Tencent’s blockchain (https://cloud.tencent.com/product/tbaas, accessed on 1 March 2020) is adopted as the blockchain infrastructure, and the underlying platform is implemented by Hyperledger Fabric (https://www.hyperledger.org, accessed on 1 March 2020). The system takes the Byzantine fault tolerance algorithm (BFT) [47] as the consensus protocol.

In Blockchain data management layer, it realizes the storage and management of business data of the platform through blockchain technology. The operation management module realizes the contract management in the operation management process, the management of equipment manufacturers, the binding of equipment to IoT cards, the management of equipment quotas in each project, and the import function after the equipment is shipped. The operation management platform calculates the operation timing of each project or device and opens and closes on time. The platform management module realizes the basic parameter configuration function for each subsystem, including non-functional management such as unified log management. The user management module manages the users in each sub-system, including role management and authority management, etc.

In the application layer, we implement the digitalization of livestock management in the whole life cycle, an intelligent support platform for regulators and insurers, and automated process management of livestock insurance. The estrus monitoring module determines whether a cow is in estrus by analyzing the device data, and actively pushes the information of the cow bound by the estrus device to the application system after estrus. The breeding management module realizes the management of basic information. The environmental monitoring module provides the environmental monitoring functions of the ranch and barn. Through this module, the administrator can automate the control of fans, shade curtains, and automatic water-filling filler systems.

### 3.2. Functionalities

#### 3.2.1. Livestock Asset Identifying

**Figure 3:**
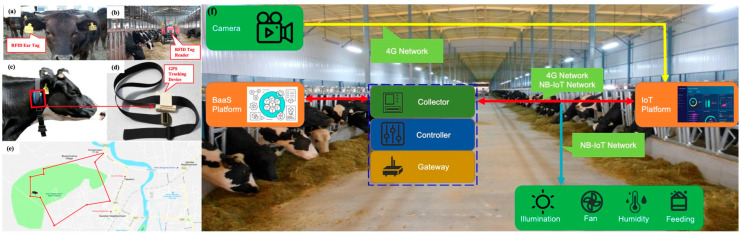
Ear Tag, Global position service Locator, Electronic Fence, and Smart Livestock Shed: (**a**) RFID ear Tag; (**b**) RFID Tag Reader; (**c**,**d**) GPS Tracking Device; (**e**) electronic fence; (**f**) internal structure of shed.

Radio frequency identification technology is widely used in various kinds of scenarios. In order to reduce the harm to livestock and the risk of radio frequency identification tag destruction by them, we use radio frequency identification-based electronic ear tags in this system (as shown in Figure 3a) to enable livestock identity management. In addition, a radio frequency identification tag can be used for inventory tracking (as illustrated in Figure 3b). The data stored inside a radio frequency identification tag cannot be changed or lost. If there are any damages to a radio frequency identification tag or attempts of insurance fraud, alarms will sound.

#### 3.2.2. Livestock Positioning

The global position service locator is worn on the neck of livestock (as shown in Figure 3c,d) to prevent livestock from becoming lost. It regularly collects information (e.g., grazing activities) and uploads it to the backend platform. Figure 3f shows the functions of a history track inquiry (such as activity mileage, grazing time, etc.) and electronic fence setting. When the livestock exceeds the pre-set range (as shown in Figure 3e), the system will remind the person in charge; and coincidently, the instant location of the livestock will be sent to the mobile devices for further actions. The global position service locator allows us to instantly obtain the location information of livestock and is also capable of estrus detection, which can carry out dynamic intervention for the breeder to improve the pregnancy rate.

#### 3.2.3. Indoor Environment Sensing for Livestock Shed

**Figure 4:**
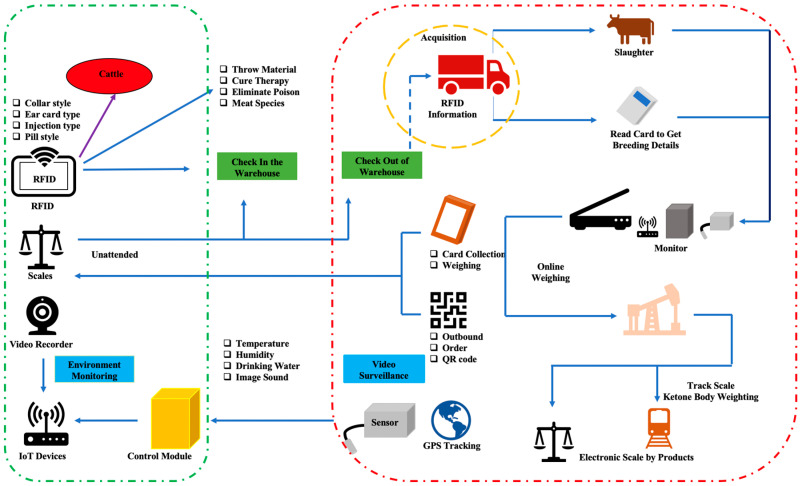
Process Traceability of Livestock Production.

In this solution, a large number of wireless sensors (e.g., temperature sensor, humidity sensor, etc.) are deployed in a livestock shed for environment detection. The air temperature, humidity, light, CO_2_ concentration, sulfide, ammonia, and other data in the shed will be periodically collected and transmitted to the IoT platform. According to the analysis of obtained data, further actions (such as opening/closing fill lights, fans, humidification, water & feed, etc.) will be taken to control the equipment (as illustrated in Figure 4). To evaluate the reliability of the received data from various sensors, the following model is also proposed. The frequency of the interaction between an IoT device “*X*” and the system is defined as:

(1) $$f_{X}=\frac{I_{X}}{I_{X,t}}$$

where *I_X* is the number of interactions between device “*X*” and the system in a certain time period; *I_*(*X*,*t*) is the total number of interactions of device “*X*” in a certain time period. Meanwhile, it is essential for the system to grade the credibility of device “*X*” when it interacts with the system. Such credibility can be expressed as:

(2) $$S_{X}=f_{X}\cdot \left(\tau \cdot S_{X\left(old\right)}+\left(1-\tau \right)\cdot S_{X\left(new\right)}\right)$$

where *S_*(*X*(*old*)) is the credibility rating of “*X*” in the previous interaction; *S_*(*X*(*new*)) is the credibility rating of “*X*” in the last interaction; and τ is the time function. This time function of trust degree evaluation can be expressed as:

(3) $$\tau =e^{-\mu \left(t_{new}-t_{old}\right)}$$

where *t_old* is the time of the previous interaction; *t_new* is the time of the last interaction; and *μ* is the time decay factor. Thus, the credibility of the system on device “*X*” can be expressed as:

(4) $$S_{X}=f_{X}\cdot \left(e^{-\mu \left(t_{new}-t_{old}\right)}\cdot S_{X\left(old\right)}+\left(1-e^{-\mu \left(t_{new}-t_{old}\right)}\right)\cdot S_{X\left(new\right)}\right)$$

#### 3.2.4. IoT-Enabled Fine-Grained Management

We develop a group of functions to support the fine-grained management of livestock. The functions of hardware management devices include video surveillance, network infrastructure, sensors, etc. Document management refers to the livestock identity files and full cycle records of livestock (e.g., birth, growth, medication, estrus, etc.). Breeder management refers to the management of livestock breeders, recording the assignment details and attendance of the breeders in each task. The function of disease management detects the healthy status of livestock and records their diagnostic history. Accurate analyses and appropriate actions could effectively prevent the large-scale spread of diseases. Schedule management refers to the task arrangement of herd transfer & slaughter, breeding, calving, weighing, milk quantity monitoring, etc. The farmer makes a schedule on the system and assigns tasks to each worker. Accordingly, the workers execute tasks and record the work details in the mobile app. The function of a breeding decision recommendation provides dynamic advice for the breeder to execute feeding tasks.

#### 3.2.5. Process Traceability of Livestock Production

Figure 4 presents how a radio frequency identification tag, worn on the livestock since its birth, is used in the process of breeding, slaughtering, and processing. The slaughterhouse reads the radio frequency identification tag of each livestock to confirm that the livestock animal is healthy and properly vaccinated before it can be slaughtered and delivered to the supermarket. In transport, the cargo driver checks the data of a radio frequency identification tag and confirms the information forwarded from previous procedures. Meanwhile, logistic information will be recorded by the system automatically in the background and the radio frequency identification tag information will be updated accordingly. After receiving the livestock products, the distributor will check and double-confirm the radio frequency identification tag information. Finally, the system will automatically generate the correspondent traceability code (e.g., QR code) for a livestock product. By scanning the traceability code, consumers can easily check the historical records of a livestock animal. Moreover, the traceability code can also be used for regulatory purposes on food quality and subsidy collection.

### 3.3. Blockchain-Driven Process of Livestock Insurance

**Figure 5:**
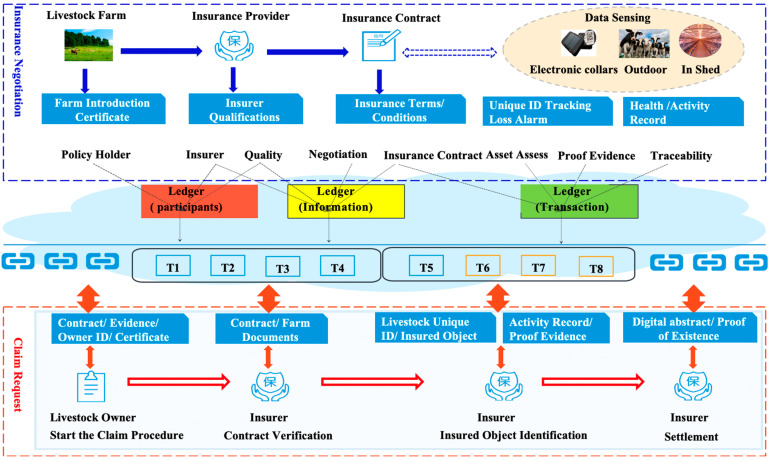
Design Rationale of Shared Ledger-based Storage.

The system realizes the digital management of livestock identification. During the collateral guarantee process, the system provides insurance agencies with instant video images and livestock identification, which can be used as a basis for inventory tracking. The identity information, inventory information, and weight information collected by IoT sensors are saved to the blockchain network to ensure authenticity (as shown in Figure 5).

We calculate the hash value for the business data and then put the information of the calling function plus the chain data into the transaction structure. After the data is processed before the signature, a hash is performed on that data, and the hash is signed. The hash is a string of values bound to the data, and tampering with the data will cause the hash value to change, so it is inherently tamper-proof. Signature signing is verified by an asymmetric encryption method that can confirm that the sender holds the corresponding private key through the public key and signature information without revealing the sender’s own private key. The chain is formed by connecting the previous block hash and its own hash, while modifying the content of any block in the chain will make the previous block hash and the modified hash of the latter block different so that the block is tamper-proof. After the completion of the consensus phase, the blocks of each node remain consistent. At this time, the business data is recognized and traceable by each node.

The system only needs to record the transaction hash of the deposited business and query through the transaction hash when reading. Both the creation of smart contracts and invocation of smart contracts go through the consensus process, so the output of the final invocation of smart contracts is also the same. The result of the completed contract invocation is written to a contract’s state database, which not only contains the latest state but also the historical state for easy traceability and query. After the logic of a smart contract is processed, the state Merkle tree is modified. If the execution of the operation of contract logic processing fails, the modifications to the state Merkle tree will also be reversed, and the data of the contract will be rolled back to the historical data before the invocation.

Once the insurance application for livestock has been submitted, information on the ear tag, ear tag clamp, location, and operator will be transmitted to the insurance company for consideration. In such a way, the identity of the insured livestock can be confirmed both by the insurance company and the livestock owner. In the claim stage, the ear tag information is read by the terminal to check the consistency of the identity of the insurance subject and realize the identity confirmation through a comparison with the livestock information registered in the system and the corresponding one recorded on the claim form.

During the negotiation stage of an insurance contract, basic information, and qualifications of each party involved in an insurance contract will be settled in the contract terms. The information on the insured livestock assets (as shown in Table 1) will also be written into the contract terms and conditions. These will be appended to the data blocks that have been once confirmed by multiple parties. Additionally, specific applications require users to share information with insurers to assess risk and insurance contract pricing. However, sharing data can compromise user confidentiality. As a trade-off, we store the user’s information on a local device, with the hash value of the data stored in the blockchain.

In the insurance claim, after a policyholder submits a claim, the insurance company starts the process to handle this claim. The insurance company instantly retrieves the record of the insured livestock (e.g., health status, location information, etc.) through the system, and compares and verifies it with the submitted information. The policyholder will receive compensation once the insurance company completes the verification process. Finally, the insurance company sends the claim receipt and claim settlement to the policyholder. The claim process is completely based on a blockchain platform containing the whole records of the claim process so that if any dispute occurs, the claim process can be traced through this system. All the business processes regarding livestock insurance are implemented through smart contracts. There are 5 types of smart contracts. We introduce these contracts in detail below.

#### 3.3.1. Contract Negotiation Transaction (CNT)

**Figure 6:**
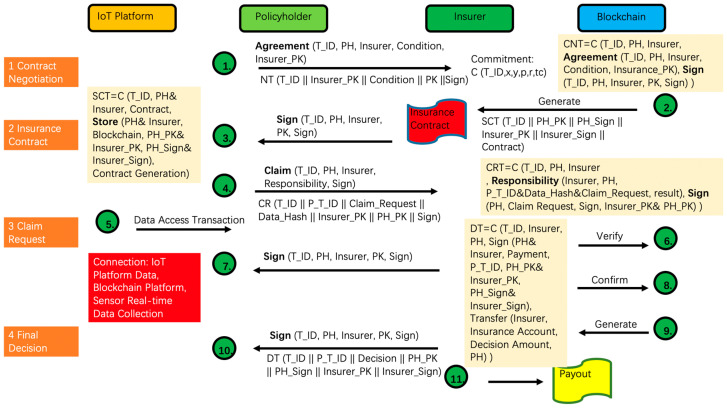
Smart Contract-Based Business Process of Livestock Insurance.

A policyholder can negotiate with the insurer about the terms and price of the insurance policy. As shown in Figure 6, “T_ID” in the CNT refers to the hash value of the transaction and acts as the unique transaction identifier. “Terms” refers to the hash values of insurance terms (e.g., rate) proposed by the policyholder. The policyholder must keep a local copy of the insurance term, and both the policyholder and insurer can execute multiple CNTs until a final agreement is reached.

#### 3.3.2. Insurance Contract Signing Transaction (ICST)

Insurance contract signing is a multi-signature transaction. It is noteworthy that the identity of a policyholder is known to the insurer, albeit remaining anonymous to other participants. The insurer stores the hash value of the contract document. In this system, the IoT data is accessible to the blockchain platform, so that we can realize real-time data validation through a large number of sensors. The policyholder creates a genesis transaction for each sensor by populating the transaction with the T_ID. The insurer also saves the record regarding the new customer in its own platform including the term, conditions, payment, smart contract ID, IoT device, and policyholder_public_key. Insurers can retrieve the contract details in the blockchain. Meanwhile, policyholders can also submit a claim to the insurer when applying for a benefit.

#### 3.3.3. Claim Request Transaction (CRT)

In the claim request transaction, P_T_ID refers to the previous transaction by the policyholder, which associates the transaction collections by the policyholder. The “claim_request” contains the details of the claim application. The policyholder can exchange the data from an IoT device with the insurer to facilitate liability determination. Then, the policyholder signs the transaction and populates the “sign” field. Upon receiving a claim request, the insurer needs to verify the policyholder’s account with the “policyholder_public_key”. It ensures that the account exists and the signature is legitimate. Subsequently, the insurer accesses data from an IoT device, which can be defined as a data access transaction (DAT).

#### 3.3.4. Data Access Transaction (DAT)

DAT is also a multi-signature transaction. A policyholder validates the request and grants the insurer the privilege to access the required data. The insurer could initiate a DAT to obtain permission for accessing the data when the data are only available in the cloud. Once the data is received, the insurer could compare the hash value (i.e., the received data) and the hash value in the blockchain to verify its integrity.

#### 3.3.5. Payout Transaction (PT)

When the final decision is made, the insurer creates a payout transaction (PT). PT is a transaction that requires multiple signatures if both the insurer and policyholder can accept the final decision. Once the transaction is approved, the system will transfer the claim amount specified in the “decision” from the insurer’s account to the policyholder’s account. The records of CRT and PT stored in the blockchain can be easily retrieved, verified, and adopted as valid evidence for a dispute.

## 4. Results

**Figure 7:**
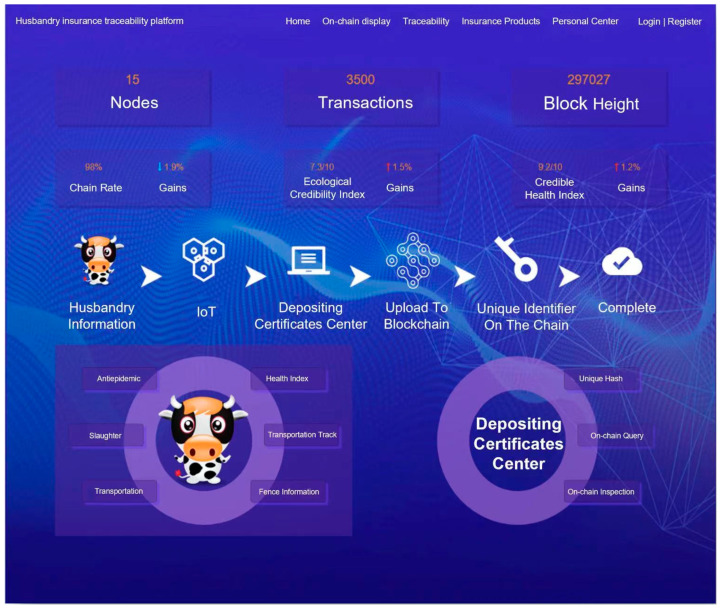
Operational Dashboard of Blockchain Platform.

**Figure 8:**
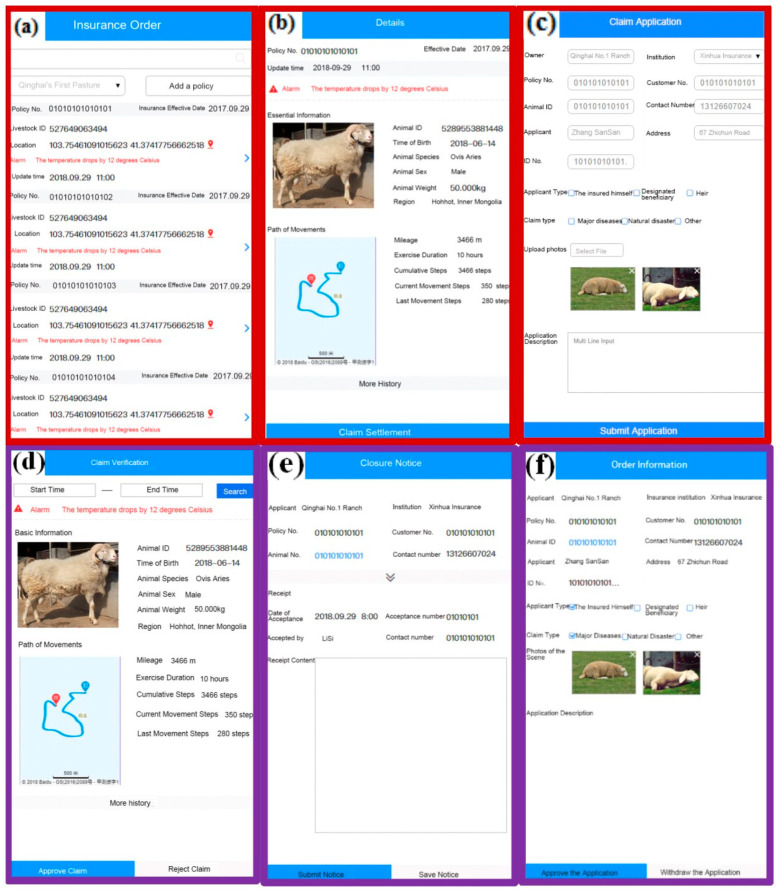
Snapshots of (**a**) Insurance Order List, (**b**) Order Details, and (**c**) Claim Application in Policyholder’s Interface; and (**d**) Claim Verification Details, (**e**) Closure Notice, & (**f**) Order Information in Insurer’s Interface.

The following are snapshots of the system running outcomes: Figure 7 shows the operation dashboard of the blockchain network. Figure 8a–c show respectively the process of an insurance company to receive claim applications, verify the insured assets, and approve claim applications. Figure 8d–f displays the claim verification details, closure notice, and order information in an insurer’s interface, respectively. Technically, the system provides a decentralized data management mechanism, which not only efficiently solves the problem of data exchange and data verification among different participants but also naturally builds trust among the participants for livestock management. It significantly improves the business operation efficiency of livestock insurance and the enthusiastic mindset of ranchers to accept livestock insurance.

The system has been adopted in a number of farms in China. At present, there are more than 300,000 registered livestock animals, with a total of more than 350,000 insurance contracts and over 20,000 insurance claims settled. The success of introducing BCT to livestock insurance allows the insurance industry to rethink the current insurance model. The use of blockchain and smart contracts can significantly reduce the cost of premium payments and insurance claims, and a personalized peer-to-peer insurance model will become possible in the future. Especially in recent years, the sharing economy has put forward new requirements for the insurance business. In these emerging business scenarios, the underlying business model and business regulations can be quickly implemented and verified in the smart contract code. This approach ensures effective compliance with the rules and provides more alternative engagement models for their policyholders. In addition, privacy protection has become a growing concern in the development of information technology. However, BCT can ensure peer-to-peer personal information exchange and identity authentication in the insurance business process, thereby safeguarding personal privacy, reducing identity and claims fraud, and increasing the confidence of business participants.

In insurance products involving personal injury, due to a lack of medical and other information related to the insured by Internet insurance institutions, claims need to be settled with the assistance of a large amount of invoices, medical records, diagnoses, and other information provided by the insured, resulting in a long claim settlement cycle and poor user experience. Based on blockchain technology, medical records can be protected by encryption and conditionally shared among medical service providers and with the outside world, which is especially important for internet insurance institutions. Relying on blockchain technology, medical insurance products can solve the problem of poor product experience caused by opaque information to both institutions and customers through effective information sharing. Meanwhile, for property and casualty insurance products, a shared distributed ledger can guarantee the authenticity of the insurance subject matter, and smart contracts can be automatically executed in case of accidents, thereby greatly improving the efficiency of property and casualty insurance policy processing.

In one-year continuous tracking of platform operation, we collect a number of feedback from farmers. Most of these farmers confirmed the effectiveness of the proposed system. e.g., these farmers reported that the system let them grasp the growth of livestock in real-time. It can efficiently realize the automatic management of pastures, reduce labor costs and improve production efficiency. On the other hand, farmers can grasp the real-time location information of each livestock during the grazing process to avoid economic losses caused by livestock loss. The regulatory reported that the system provided effective supervision of meat production, processing, transportation, and sales in all aspects to a certain extent. Farmers also reported that this system can effectively prevent the spread of diseases in livestock. When there is a livestock disease, the system can promptly alert the background management staff for effective prevention and management to avoid the spread of the epidemic.

## 5. Discussion

### 5.1. Comparison of This Study with Existing Studies

The current study aligns with the prior literature on blockchain applications in the livestock industry. Satamraju developed a blockchain-based platform for livestock traceability in China [41]. Vahdati et al. developed a blockchain-based platform for livestock health management in China, which found that the platform improved disease prevention and control, data sharing, and decision-making [18]. Furthermore, this study also aligns with the previous literature on blockchain applications in the insurance industry. e.g., Dey et al. developed a blockchain-based platform for insurance claims management in China, which aimed to address the challenges of data reliability, transparency, and fraud detection [48] Viriyasitavat et al. proposed a study focused on the application of blockchain technology in the traceability of livestock products [49]. These studies focused on the application of blockchain technology in pig/sheep breeding and management [34,42,50].

However, this study differs from existing blockchain applications in the livestock industry in terms of focus and scope. Most prior studies focused on livestock traceability or health management, whereas this study focused on livestock insurance. Additionally, most prior studies were limited in scope, whereas this study had a broader scope, covering many farms in China with a large number of registered livestock animals, insurance policies, and claims. The research results also differ from the prior literature on blockchain applications in the insurance industry in terms of approach and methodology. The combination of Blockchain and IoT is introduced in this study. Additionally, most prior studies used a centralized or hybrid architecture, whereas this study used a decentralized architecture.

### 5.2. Limitations to the Current Study

The research results also have some limitations that need to be addressed in future studies. Firstly, the research was conducted in China, and the results may not be generalizable to other countries or regions with different contexts and cultures. Future studies need to test the feasibility and effectiveness of the decentralized regulatory platform for livestock insurance in other countries or regions. Secondly, the research focused on livestock insurance, and the results may not apply to other types of insurance in other industries. Future studies need to investigate the applicability and effectiveness of blockchain technology in other types of insurance in other industries. Thirdly, the research used a quantitative approach, and the results may not capture the nuanced and subjective experiences of the stakeholders involved. Future studies need to use a mixed-methods approach that combines quantitative and qualitative methods to capture the complex and diverse perspectives of the stakeholders involved.

## 6. Conclusions

In conclusion, our proposed BCT and IoT-based solution for livestock insurance has demonstrated its effectiveness in addressing the challenges faced by the livestock industry in China. By providing a regulatory platform with transparent processes and reliable data, the proposed solution has enhanced the efficiency and transparency of livestock insurance. The system has been successfully implemented in many farms in China, managing over 300,000 registered livestock animals, 350,000 insurance policies, and 20,000 insurance claims. The research results have several major themes that have implications for the livestock and insurance industries. Firstly, the use of blockchain technology can address the challenges of data reliability and process transparency, which can prevent cases of insurance and subsidy fraud, which are common in the livestock industry. Secondly, the use of blockchain technology can enhance the efficiency of insurance claims management in the livestock industry. This can reduce the time and cost of processing insurance claims, which can benefit both farmers and insurers. Thirdly, the workflow proposed in this study can be used for microinsurance, which can benefit small-scale farmers who cannot afford conventional insurance. This can enhance the financial inclusion and social welfare of small-scale farmers, who are often marginalized in the livestock industry. In the future, we will focus on privacy protection while applying this system in the insurance industry, which is a critical issue that cannot be ignored in BCT-driven applications for other fields. Overall, our study demonstrates the potential of BCT to revolutionize the livestock insurance industry and provides a model for other countries looking to improve the effectiveness of their livestock insurance programs.

## Figures and Tables

**Table 1:** Properties of Records in the Proposed System.

Record	Property	Description
Livestock profile (Insured assets)	Ear tag ID	RFID device
	Breed	Type of livestock
	Gender	
	Age	
	Coat Color	
	Fattening cycle	Growth period
	Policyholder	Livestock owner
	Barn ID belonging to	
	Breeder	Person in charge
	Photo (video)	
Feed	Feeding name	
	Supplier name	Feeding supplier
	Batch information	Feeding tracking code
	Mould detection	
	Colony detection	
	Feeding time	
	Feeding method	
	Inspection records	
Disease	Diagnosis date	
	Disease name	
	Diagnosis record	
	Treatment cycle	
	Drug supplier	
	Livestock ID	
	Rest period	
	Veterinarian in charge	
Vaccination	Vaccine manufacturer	
	Manufacturer license	
	Regulatory agency	
	Livestock ID	
	Name of drug	
	Name of disease	
	Date	
	Farm area	
	Veterinarian in charge	
Production	Product name	
	Grade	
	Production date	
	Production raw material	e.g., meat, beef
	Livestock ID	Ear tag ID
	Execution standard	e.g., ISO/TS 22002-3
	Production batch information	
	Producer	
	Raw material supplier	

## Data Availability

The data used in this study is available by requesting to corresponding author.
